# Clinical Profile and Visual Rehabilitation with Mini-Scleral Device in Irregular Corneas at a Tertiary Eye Hospital : An Observational Study

**DOI:** 10.31729/jnma.9134

**Published:** 2025-07-31

**Authors:** Pankaj Ray Adhikari, Tabassum Aara, Pradeep Kumar Patel, Rajiv Ranjan Karn

**Affiliations:** 1 Contact lens Department, Biratnagar Eye Hospital, Biratnagar, Nepal; 2 National Academy of Medical Sciences, Kathmandu; 3 Research Department, Lahan Eye and Ear Care System, Biratnagar

**Keywords:** *corneal disorders*, *eye hospital*, *mini scleral lens*, *Nepal*

## Abstract

**Introduction:**

Keratoconus, Stevens-Johnson syndrome, corneal dystrophy, and corneal scar are the sight threatening conditions of the anterior eye. Optical management of these irregular corneas include use of spectacles and contact lenses like corneal lenses (GP) as well as corneo-scleral/mini scleral and scleral lenses. Mini scleral device(MSD) delivers refractive corrections to most of the irregular corneas and provide maximum comfort with proper visual rehabilitation. It also halts the corneal transplant in many cases. The purpose of this study was to determine the clinical profile andvisual outcome ofmini scleral device in different irregular corneas.

**Methods:**

The hospital based cross-sectional study was conducted between July 2022 to December 2023 in contact lens department of tertiary eye hospital of eastern Nepal. During study period total 208 eyes of 128 patients were included in the study. Eyes with different irregular cornea in which mini scleral device trial was performed were included in the study.

**Results:**

The mean age (±SD) of the patients was 21.03 (6.70) with minimum 11 years and maximum 42 years. Majority of them were male 94 (73.44%) and below 30 years of age 113 (88.28%). Majority of eyes had keratoconus 180 (86.54%). After trial of MSD, majority of the eyes 201 (96.63%) achieved normal vision (0.0-0.50 log MAR) among them 84 (40.38%) eyes had achieved 0.0 log MAR visual acuity.

**Conclusions:**

Keratoconus was found the most common irregular cornea and majority of eyes achieved normal vision with MSD.

## INTRODUCTION

Irregular corneas such as Keratoconus, Corneal scar, Steven Johnson syndrome and Pellucid marginal degeneration (PMCD) have presence of high and irregular corneal astigmatism which cause poor vision.^1^ These conditions can be managed optically with spectacle and contact lenses. Conventional RGP lens has limitation in fitting most of the irregularities so Mini scleral device (MSD), the large diameter gas permeable lens fit properly in most of the irregularities to provide maximum comfort and visual rehabilitation.^2^

These Mini scleral devices are a category of scleral lenses with overall diameters of 14 to 18 mm smaller than the 18.1 to 24 mm large scleral contact lenses.^3^ With MSD, clinicians are more comfortable in fitting and patients find care and handling more acceptable and it also halt the corneal transplant in many cases.^4,5^

The objective of this study was to find out the clinical profile and visual rehabilitation of mini scleral device in different irregular corneas.

## METHODS

This study was a hospital based cross sectional study conducted in contact lens department of Birantnagar Eye Hospital (BEH) which is a referral and tertiary eye hospital of Nepal. The Data was taken from the Electronic Medical Record (EMR) of contact lens department retrospectively from July 2022 to December 2023. All the patient’s eyes in which MSD were trialed were included in the study. The study adhered to the tenets of Declaration of Helsinki and Ethical clearance was taken from the institutional review committee of BEH with reference number: 98/2023. The routine preliminary examination for contact lens evaluation was performed followed by contact lens trial by an optometrist trained in contact lens care.^6^ The initial examination involved obtaining a comprehensive patient history and conducting a preliminary assessment. This included measuring uncorrected vision at both distance and near, as well as various ocular measurements such as horizontal corneal diameter, vertical palpebral aperture, pupil size under both bright (photopic) and dim (mesopic) lighting conditions to assist in determining the appropriate lens design and type. Objective Refractions were performed using both Auto refractometer and Retionscope and, subjective refractions were performed using a trial frame using loose trial lenses preferred for many younger contact lens wearers, as it may reduce accommodation compared to a phoropter. A slit-lamp examination was also conducted to evaluate the conditions of eyelids, conjunctiva, cornea, and tear film. Corneal topography was also performed which help to guide the selection of the initial trial lens, along with other supplementary tests as needed. After the initial examination and contact lens trial, mini scleral devices ranging from 14.5 to 16 mm in diameter were used for irregular corneas where a proper fit and satisfactory visual acuity could not be attained with other types of contact lenses. The base curve of MSD was selected by observing the severity of irregular cornea (keratometry reading and sagittal height) and fitting guidelines provided by the manufacturing company of MSD. Demographic profile such as age, gender, laterality, Uncorrected Visual Acuity (UCVA) and Best Corrected Visual Acuity (BCVA) with MSD, manifest refraction, type and amount of refractive error were taken for the analysis by entering in excel sheet and analysis was performed by using SPSS (Statistical Package for the Social Sciences) software version 23.

UCVA and BCVA was measured using CAT (Comprehensive Vision Tester) vision LCD chart developed by Auro lab. Refractive error was classified as following criteria.^7^

A) Hyperopia: Refractive error of at least +0.5 D and above. This was further classified as Low (+0.50D to +3.0D), Medium (+3.0D to <+6.0D) and High (more than +6.0D)
B) Myopia: Refractive error at least -0.5 D and below. This was further classified as Low (-0.50D to -3.0D), Medium (-3.0D to <-6.0D) and High (more than -6.0D)
C) Astigmatism: astigmatism was classified as Simple Hyperopic Astigmatism (SHA), Simple Myopic Astigmatism (SMA), Compound Hyperopic Astigmatism (CHA), Compound Myopic Astigmatism (CMA), and Mixed Astigmatism (MA).

The Visual Acuity was categorized as per WHO (World Health Organization) guidelines. (0.0 to 0.5 log MAR -Normal), (0.6 to 1.0 log MAR-Visual Impairment), (1.0 to1.3 log MAR-Severe Visual Impairment), (<1.3 log MAR to NPL-Blind).^8^

Keratoconus was also classified according to Collaborative Longitudinal Evaluation of Keratoconus study (CLEK) classification: mild (steep keratometry [K] < 45 diopters [D]), moderate (steep K between 45 D and 52 D), and severe (steep K >52 D).^9^

## RESULTS

During the study period, total 208 eyes of 128 patients were analyzed. The mean age of the patients was 21.03±6.70 years with a minimum 11 years and a maximum 42 years. There were 94 (73.40%) male particpants and 116 (90.6%) particpants were below the age of 30 years. Keratoconus was observed in 180 (86.60%) eyes (Table 1). Among Keratoconus, 129 (71.70%) eyes were of male patients.

**Table 1:** Demographic profile of patients undergoing visual rehabilitation with mini scleral device in irregular corneas (n=208 eyes of 128 patients).

Demographic and Clinical Characteristics	n(%)
Gender	
Male	94(73.44)
Female	62(48.44)
Age group (years)	
11-19	62(48.44)
20-29	51(39.84)
30 and above	15(11.72)
Diagnosis (n=208 eyes)	
Keratoconus	180(86.54)
Corneal scar	15(7.21)
Post PK	8(3.85)
PMCD	5(2.40)

**Table 2:** Clinical findings of patients undergoing visual rehabilitation with mini scleral device in irregular corneas (n=208 eyes of 128 patients).

Indicators	Mean ± SD	Minimum	Maximum
Visual Acuity (log MAR)			
UCVA in both eye	1.04±0.45	0.20	1.60
Right eye	1.10±0.45	0.20	1.80
Left eye	0.98±0.45	0.20	1.60
BCVA in both eye with glass	0.64±0.48	0.00	1.60
Right eye	0.66±0.47	0.00	1.60
Left eye	0.62±0.49	0.00	1.60
BCVA in both eye with MSD	0.16±0.21	0.00	1.60
Right eye	0.18±0.21	0.00	1.40
Left eye	0.16±0.20	0.00	1.60
Max Sim K reading (Diopter) both eye	57.50±8.80	42.96	87.20
Right eye	58.29±8.70	43.34	82.01
Left eye	56.73±8.90	42.58	92.39
Astigmatism (Diopter) both eye	5.91±3.60	0.15	22.33
Right eye	6.28±3.80	0.24	22.33
Left eye	5.54±3.34	0.15	18.83
Sagittal Depth (SAG) of mini scleral devices (mm) both eye	4.51±0.43	3.98	6.15
Right eye	4.55±0.47	3.98	6.15
Left eye	4.47±0.38	3.98	5.85

**Table 3:** Patient distribution between BCVA with glass and MSD within age group and gender (n=208 eyes of 128 patients)

Age / Gender	BCVA with Glass 0.0-0.5 log MAR n(%)	BCVA with Glass 0.6-1.6 log MAR n(%)	BCVA with MSD 0.0-0.5 log MAR n(%)	BCVA with MSD 0.6-1.6 log MAR n(%)
Age(years)				
11 to 19	48(23)	46(22.11)	92(44.23)	2(0.96)
20 to 29	47(22.59)	40(19.23)	82(39.42)	5(2.40)
≥30	18(8.65)	9(4.32)	27(12.98)	0(0)
Gender				
Male	90(43.27)	61(29.32)	147(70.67)	4(1.92)
Female	23(11)	34(16.34)	54(25.96)	3(1.44)

BCVA = Best Corrected Visual Acuity; MSD = Mini Scleral Device; log MAR = Logarithm of the Minimum Angle of resolution

The Mean maximum Simulated Keratometry (Max Sim K) reading was 58.29 ±8.70 diopter in right eye and 56.73±8.90 diopter in left eye. Mean astigmatism was 6.28 ±3.80 diopter in right eye and 5.54 ±3.34 diopter in left eye. The mean sagittal depth of mini scleral contact lens used was 4.55±0.47 mm in right eye and 4.47 ±0.38 mm in left eye (Table 2).

**Figure 1:**
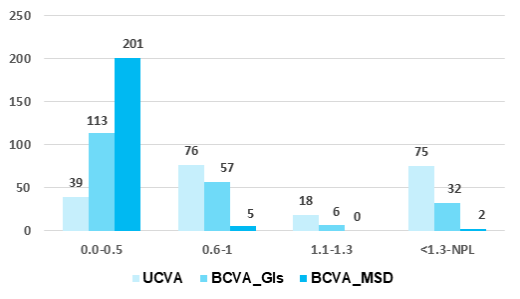
Visual status of patients undergoing visual rehabilitation with mini scleral device in irregular corneas (n=208 eyes of 128 patients). UCVA=Uncorrected Visual Acuity; BCVA=Best Corrected Visual Acuity; Gls=Glass; MSD=Mini Scleral Device; NPL=No Perception of light

Normal vision (0-0.5 log MAR) was achieved in 201 (97 %) eyes. Among them, 84 (40.60%) eyes had 0.0 log MAR visual acuity (Figure 1).

There were 113 patients in BCVA with glass in both eye category with log MAR between 0.0-0.5 and with log MAR between 0.6 and 1.6. Similarly, there were 201 patients in BCVA with MSD in both eye category with log MAR between 0.0-0.5 and 7 with log MAR between 0.6 and 1.6 (Table 3).

There was no any significant difference in BCVA with glass and MSD with age group. There was also no significant difference in BCVA with MSD and gender but significant difference in BCVA with glass with gender (P=0.01) (Table 3)

**Table 4:** Patient distribution as per severity of Keratoconus and Sagittal depth (SAG) of MSD (n=180 eyes of 90 patients).

SAG of MSD up to 5.0 mm n (%)		
Severity of Keratoconus	up to 5.0 mm n (%)	>5.0 mm n (%)
Mild to Moderate	48 (26.67)	3 (1.67)
Severe	106 (58.89)	23 (12.77)

SAG = Sagittal Depth; MSD = Mini Scleral Device

BCVA with glass in both eyes were found between 0.0-0.5 log MAR in 113 (54.27%) eyes and between 0.6-1.6 log MAR in 95 (45.66%) eyes. Similarly, BCVA with MSD in both eyes were found between 0.0-0.5 log MAR in 201 (96.63%) eyes and between 0.6 -1.6 log MAR in 7 (3.36%) eyes (Table 3).

The Sagittal depth of MSD was used up to 5.0 mm in 154 (85.56%) eyes and more than 5 mm in 26 (14.44%) eyes (Table 4).

## DISCUSSION

In this study we are reporting feasibility and efficacy of mini scleral contact lenses in correcting irregular corneas optically which could not be corrected with spectacles or smaller diameter corneal GP lenses. According to our results, the best corrected visual acuity was dramatically increased with MSD in these patients. We found Keratoconus as the major corneal irregularity in most of the eyes and more in male compare to female. Majority of the eyes achieved normal vision with MSD. The mean difference of visual acuity was also significantly improved with MSD as compare to glass.

Biratnagar eye hospital is a tertiary eye care hospital providing high volume and high quality eye care services with affordable cost in eastern Nepal with different sub specialty clinics including contact lens which is a referral clinic for the management of different irregular corneas from eastern part of Nepal as well as the neighboring countries India, Bhutan and Bangladesh. The hospital is managed by Lahan Eye and Ear Care System (LEECS) former Eastern Regional Eye Care Programme (EREC-P) under the ageis of Nepal Netra Jyoti Sangh (NNJS).^10^

Non-surgical management of an irregular cornea poses significant clinical challenges, especially when visual rehabilitation is the primary goal. Conventional glasses and soft contact lenses are generally ineffective in such cases, as they have limited ability to correct the irregular corneal surface effectively and often fail to provide satisfactory visual improvement. Regular corneal gas permeable (GP) lenses can provide good visual outcomes in the early stages of many corneal disorders, as they create a tear reservoir between the anterior corneal surface and the lens's posterior surface, helping to correct various corneal irregularities^11^ but as corneal irregularities worsen in conditions like advanced Keratoconus, corneal scarring, and Pellucid marginal degeneration, corneal GP lenses may no longer fit properly, leading to compromised visual acuity. In such cases, mini-scleral or scleral lenses are the best option for better visual rehabilitation.^12^

Few years ago, scleral and mini-scleral lenses were rarely available in developing countries like Nepal primarily due to their high cost and other factors. However, these lenses are now widely available in most of the tertiary eye hospitals across Nepal. Due to reduced cost and better visual rehabilitation, their popularity has steadily increased in recent decades.^13^ Many studies suggested that, the low diameter mini scleral lens were the good alternative of visual rehabilitation where corneal GP and spectacles not provide satisfactory results in many of irregular corneal surface.^9,14,15^ In our study, patients demonstrated improved corrected visual acuity with mini scleral lenses compared to spectacles. This enhancement is likely due to the presence of substantial irregular astigmatism in which mini scleral lenses are better equipped to correct.

In this study, we identified keratoconus as the leading cause of corneal irregularities, predominantly affecting males, aligning with the findings of Suh SY et al.^16^ The average log MAR visual acuity improved significantly from 1.04 ± 0.45 to 0.16 ± 0.21 with the use of MSD, consistent with results reported by Suh SY et al.^16^ Additionally, the mean corneal astigmatism observed in our study was 5.91D ± 3.57, which is higher than the values reported by Montalt, J.C .et al and Suh SY et al.^15,16^ This could be attributed to the higher proportion of moderate and severe Keratoconus cases within the study cohort. The average sagittal depth of the MSD was measured 4.51 mm ± 0.42, which was lower than the value reported in the study by Kim S et al this may be explained by the predominance of severe keratoconus cases included in this study. ^17^

This was a relatively larger sample size study as compared to other similar studies. Due to the retrospective nature of the study, data of mean wearing time, average number of lens trials, re-prescriptions required for optimal fitting, contrast sensitivity, ocular aberrations, and quality of vision and life with MSD could not be retrieved. Therefore, further prospective studies are warranted to explore these parameters in greater detail.

## CONCLUSIONS

Keratoconus emerged as the predominant form of corneal irregularity in our study. Majority of eyes attained normal functional visual acuity with the use of MSD.

## Data Availability

The data are available from the corresponding author upon reasonable request.

## References

[1] Rathi VM, Mandathara PS, Taneja M, Dumpati S, Sangwan VS (2015). Scleral Lens for Keratoconus: Technology Update. Clinical Ophthalmology.

[2] Shorter E, Harthan J, Nau CB (2018). Scleral Lenses in the Management of Corneal Irregularity and Ocular Surface Disease. Eye Contact Lens.

[3] Jorge LA, Alfredo VE, Dez PS, Garca PP, Garca MLD, Maldonado M (2015). Keratoconus Management Guidelines. Int J Keratoconus Ectatic Corneal Dis.

[4] Ye P, Sun A, Weissman BA (2007). Role of Mini-Scleral Gas-permeable Lenses in the Treatment of Corneal Disorders. Eye Contact Lens.

[5] Fateme A, Ahmad K, Mahmoud JB (2012). Use of Miniscleral Contact Lenses in Moderate-to-Severe Dry Eye. Cont Lens Anterior Eye.

[6] Vazirani J, Basu S (2013). Keratoconus: Current Perspectives. Clin Ophthalmol.

[7] Althomali TA (2018). Relative Proportion of Different Types of Refractive Errors in Subjects Seeking Laser Vision Correction. Open Ophthalmol J.

[8] Khawaja AP (2013). The EPIC-Norfolk Eye Study: Rationale, Methods and a Cross-Sectional Analysis of Visual Impairment in a Population-Based Cohort. BMJ Open.

[9] Wagner H, Barr JT, Zadnik K (2007). Collaborative Longitudinal Evaluation of Keratoconus (CLEK) Study: Methods and Findings to Date. Cont Lens Anterior Eye.

[10] Lahan Eye and Ear Care System.

[11] Gemoules G (2005). Therapeutic Effects of Contact Lenses after Refractive Surgery. Eye Contact Lens.

[12] Romero-Jimnez M, Flores-Rodrguez P (2013). Utility of a Semi-Scleral Contact Lens Design in the Management of the Irregular Cornea. Cont Lens Anterior Eye.

[13] Porcar E (2019). Impact of Corneoscleral Contact Lens Usage on Corneal Biomechanical Parameters in Keratoconic Eyes. Eye Contact Lens.

[14] Alipour F, Rahimi F, Hashemian MN, Ajdarkosh Z, Roohipoor R, Mohebi M (2016). Mini-Scleral Contact Lens for Management of Poor Visual Outcomes after Intrastromal Corneal Ring Segments Implantation in Keratoconus. J Ophthalmic Vis Res.

[15] Montalt JC (2018). Visual Quality with Corneo-Scleral Contact Lenses for Keratoconus Management-Part Ii. Cont Lens Anterior Eye.

[16] Suh SY, Lee JH, Lee SU, Park YK, Lee JS, Lee JE (2016). Fitting the Mini scleral Contact Lens in Patients with Corneal Abnormalities. J Korean Ophthalmol Soc.

[17] Kim S, Lee JS, Park YK, Lee SU, Park YM, Lee JH, Lee JE (2017). Fitting miniscleral contact lenses in Korean patients with keratoconus. Clin Exp Optom.

